# Gender Disparity in Cardiovascular Disease in the Era of Precision Medicine

**DOI:** 10.1016/j.jaccas.2023.101985

**Published:** 2023-10-04

**Authors:** Naeimeh Hosseini, Thomas E. Kaier

**Affiliations:** a Cardiology Department, St Thomas’ Hospital, Guy’s and St Thomas’ NHS Foundation Trust, London, United Kingdom; b King’s College London BHF Centre, The Rayne Institute, St Thomas’ Hospital, London, United Kingdom

**Figure:**
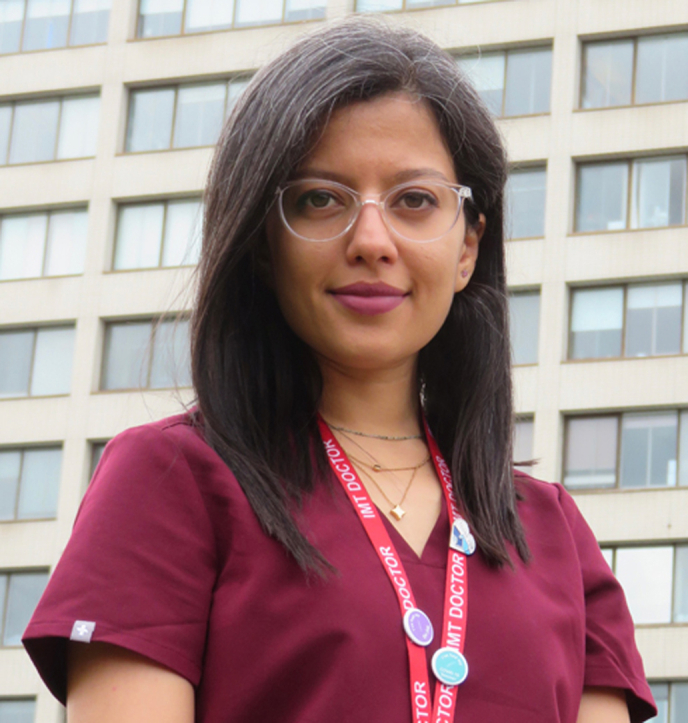

Cardiovascular disease (CVD) has remained the leading cause of mortality and morbidity in women, accounting for over one-third of all deaths in this population. However, according to the Lancet CVD Commission publication in 2019, CVD has remained “understudied, under-recognized, underdiagnosed and undertreated” in women around the world.^1^ Have we achieved the precision (and there is no precision without equality) in health care outcomes that the advent of the 21st century has promised to deliver?

Numerous studies have shown differences between men and women in cardiovascular risk factors, pathophysiology, clinical manifestations, response to treatment, and eventually, outcomes in CVD. However, most evidence is derived from trials with predominantly male participants. A paucity of gender-specific risk stratification tools and guidelines results in the delayed diagnosis of CVD in women—often with a higher burden of symptoms and comorbidities at the time of presentation and less likely to benefit from effective treatment resulting in worse outcomes. For instance, in patients with symptoms of ischemic heart disease, the prevalence of nonobstructive coronary artery disease is 2-fold higher in women. Still, women are less likely to receive guideline-intended medical treatment and less likely to undergo early angiography. Subsequently, the diagnosis of ischemic heart disease in women with nonobstructive CAD is often delayed, and the risk of adverse outcomes is higher.^2^

This also holds true for valvular heart disease. Whereas women more frequently develop diseases of the mitral valve, men are more likely to have aortic valve disease and infective endocarditis.^3^ Moreover, there are gender-specific differences in pathophysiology, ventricular response to pressure, and volume overload, which subsequently lead to different manifestations of valvular heart disease. For instance, whereas men develop more calcified aortic valves, hemodynamically severe aortic stenosis (AS) is seen at a lower degree of calcification in women, possibly caused by a higher burden of valvular fibrosis in women.^3^ Women with AS also undergo concentric left ventricular remodeling associated with low-flow (<35 mL/m^2^) low-gradient AS and increased mortality. Gender-specific thresholds are therefore important to define severe AS. But even when diagnosed, women are less likely to be considered for surgical valve replacement, and those who undergo surgical aortic valve replacement are often older with higher frailty and comorbidities and higher postprocedure mortality.^3^ Not surprisingly, current guidelines on disease severity, cardiac dimensions, and thresholds for intervention were developed from trials where women were under-represented, and more gender-specific research will be required to increase the identification of women who will benefit from early intervention.

Introduced decades ago, cardiac troponin is used routinely for assessment and prognostication of CVD disease, and yet, gender differences are neither exhaustively studied nor fully understood. Advances in the quantification of high-sensitivity cardiac troponin (hs-cTn) have helped to identify differences in troponin concentration in both genders: recent studies have consistently shown lower cardiac troponin concentration in women compared with men in both healthy subjects and those with established cardiovascular disease. In a study of over 19,500 individuals (58% women), the threshold associated with a doubling of cardiovascular risk was lower in women compared with men.^4^ Accounting for the lower threshold, cardiac troponin concentration remained highly predictive of the future risk of CVD, with a trend toward better discrimination in women—although the difference between genders was attenuated after adjusting for cardiovascular risk factors and prior disease. Seeking not to waste granularity gained by the use of hs-cTn assays, it was proposed that troponin should be used as a continuous measure to risk-stratify individuals in a multifactorial approach. And if used in isolation, it seems imperative to adopt a gender-specific approach.^5^ Binary cutoffs do not stand the test of time—neither in a “snapshot” quantification of hs-cTn nor in the multiyear trajectory of change in cardiac troponin concentrations, which varies greatly between women and men. While, eg, troponin I concentrations in women lag by a decade, the relative change over 15 years is accelerated.

But, implementing gender-specific parameters will not be the panacea without further research! In the High-STEACS (High-sensitivity Troponin in the Evaluation of patients with Acute Coronary Syndrome) trial, implementing a gender-specific threshold for hs-cTnI in patients with suspected acute coronary syndrome was associated with a 5-fold increased diagnosis of myocardial injury in women, such that both genders had a similar rate of diagnosis—but at little improvement of overall outcomes. The implementation of gender-specific thresholds was associated with increased coronary angiography, revascularization, and initiation of preventive medication. Intriguingly, the rates of subsequent myocardial infarction or cardiovascular death remained unchanged in both genders.^5^ This could be attributed to lower rates of investigation and treatment in women with type I myocardial infarction compared with men, but certainty is lacking.

In the era of precision medicine, we still struggle to fully understand, appreciate, and respond to differences in the pathogenesis, pathophysiology, and natural history of CVD in women. Gender-specific data on CVD in women from many regions of the world is scant or absent, and even in developed countries, such data is affected by the under-representation of women in clinical trials and registries.^1^ Quality registries are needed to understand the natural course of the disease in women and to establish a powerful real-world data set that lays the foundation for future research. Clinical trials where women are adequately represented are also pivotal because risk factors, etiology, and comorbidities that affect outcomes in women are different. Clinical trials should be powered to identify gender-specific differences to produce reliable and effective guidelines and recommendations. Gender-specific cutoffs and indexed measures accounting for differences in body size and gender-specific biological factors should be studied and implemented whenever possible. Not only is gender-specific evidence synthesis and diversity in research important, but there is also a need to increase awareness among clinicians about undertreatment and implicit bias. Suboptimal adherence to the prescription of guideline-recommended treatment is a well-recognized issue that confers a negative impact on the outcome of CVD in women. Delayed diagnosis, under-referral, and a higher threshold to referral for invasive procedures remain major hurdles that can lead to outcome disparities.^2^ Ultimately, successful management of CVD in women relies on better diagnostic and prognostic tools tailored to the needs of individuals derived from studies with better inclusivity and diversity. Only then will we be able to infuse more of that warranted precision into the medicine we deliver.

## Funding Support and Author Disclosures

The authors have reported that they have no relationships relevant to the contents of this paper to disclose.
